# Health-Related Quality of Life after Cataract Surgery in Armenia: A Cross-Sectional Survey

**DOI:** 10.3390/healthcare11172429

**Published:** 2023-08-30

**Authors:** Tsovinar Harutyunyan, Aida Giloyan, Varduhi Petrosyan

**Affiliations:** Turpanjian College of Health Sciences, American University of Armenia, Yerevan 0019, Armenia; aida@aua.am (A.G.); vpetrosi@aua.am (V.P.)

**Keywords:** health-related quality of life, social support, visual outcome, cataract surgery

## Abstract

Cataract surgery helps to enhance visual function and improve the quality of life of cataract patients. The present study assessed visual outcomes and explored health-related quality of life (HRQoL) and factors associated with it following cataract surgery in Armenia. An interviewer-administered survey along with the ophthalmologic examination was conducted among 248 patients. It explored socio-demographic characteristics, use of eye care services, smoking status, comorbidities, and receiving and giving social support. A Short Form Health Survey (SF-36) was used to measure HRQoL. Simple and multivariable linear regression was used for the analysis. About 72.8% of examined eyes had good visual outcomes, while 17.7% had borderline outcomes. Poor visual outcomes were detected in 9.5% of the eyes. The mean composite SF-36 score for physical health was 50.8, while the mean composite score for mental health was 49.9. Gender, socioeconomic status, having a non-communicable disease, and receiving and giving tangible social support were significantly associated with SF-36 physical component in the adjusted analysis, while the variables which demonstrated significant association with the mental component included socioeconomic status, having a non-communicable disease, and giving tangible support. The visual outcome after cataract surgery in Armenian patients is below WHO-recommended standards. The quality of ophthalmological surgical care should be monitored to maximize the visual outcome in Armenian patients, with a focus on women, patients with poor socioeconomic status, and those with non-communicable diseases.

## 1. Introduction

Cataract remains the leading cause of visual impairment and blindness globally despite improved delivery of cataract surgical services in many parts of the world [1,2]. Cataract surgery is one of the most common and cost-effective surgical procedures performed worldwide [3,4]. Outcomes of the surgery are measured not only in terms of visual acuity in the operated eye or per patient but also as the ability to function and quality of life [5]. In most cases, cataract surgery substantially improves visual function and enhances the quality of life in affected patients [4,6]. 

The World Health Organization (WHO) defines visual outcome after cataract surgery as good (visual acuity less than 6/6 and equal to or better than 6/18), borderline (visual acuity less than 6/18 and equal to or better than 6/60) and poor (visual acuity less than 6/60) [7]. According to WHO recommendations, after cataract surgery, 80%, 15% and 5% of operated eyes should have a good, borderline and poor visual outcome with available correction, respectively [7]. Sometimes, however, cataract surgery may fail to improve visual function and introduce a small, but significant risk of permanently reduced vision or even death following surgery [8]. Complications such as macular edema, intraocular hemorrhages and postoperative emmetropia may take place after cataract surgery, which could impede regaining adequate visual acuity after cataract surgery [9]. The outcomes of the surgery are generally poorer in less-developed countries [5,10,11,12,13,14,15,16]. Limburg et al. reported good visual outcome in 37.8% of patients, a borderline visual outcome in 45.6% of patients and a poor visual outcome in 16.6% of patients after cataract surgery with the available standard correction in the Indian population [5], while Lau et al. found that 40.0% of cataract operated patients had good visual outcome and 4.5% of patients had poor visual outcome with the available correction in patients living in Hong Kong [16]. The main reasons for having poor or borderline visual outcomes after cataract surgery might include uncorrected aphakia and surgeon practice level [5,16]. 

Visual impairment has been shown to influence health-related quality of life (HRQoL) [17,18,19]. While there is no universal agreement on how HRQoL should be defined, some aspects are included in most explanations of this comprehensive and complex concept [20]. HRQoL is a multidimensional concept that describes the ability of a person to function well in his/her daily life and self-assessed physical, social, and mental well-being [21]. With visual impairment, quality of life may be affected by a reduced ability to conduct vision-related daily living activities or have social interaction [19]. Visual impairment is associated with depression, frustration, and anxiety, not only because of the impairment but also because of the accompanying worry that the condition may worsen [22,23]. Even mild visual impairment has been shown to be independently associated with poor HRQoL [24]. 

Several studies have investigated HRQoL in cataract patients before and after cataract surgery [25,26,27,28,29,30]. Most studies showed that patients reported significantly improved HRQoL after cataract surgery [25,26,27,28], while others found no significant difference in the mean score of SF-36 before and after cataract surgery [29,30]. The analysis of the performance of select quality-of-life measures conducted by Kaplan et al. suggested that different HRQoL measures might not be equally responsive to vision-improving interventions, such as cataract surgery, and that generic quality-of-life measures might be less sensitive to improvements than disease-targeted measures [31]. However, several recent studies have shown the suitability of generic quality-of-life measures, including SF-36, for reflecting the impact of visual impairment in cataract patients [32,33,34]. 

Social support is one of the most important contributors to HRQoL for people with visual impairments who might have feelings of being isolated from society and a decrease in social status [35,36,37]. Social support can help visually impaired people adapt and cope with vision loss [37,38,39]. Both emotional and instrumental support was shown to be important for the HRQoL of adults with visual impairment [40,41]. In contrast, other studies found that under stressful situations, receiving social support is positively linked with negative effects [42] and mental health problems [43,44]. Receiving social support can create feelings of gratitude, a threat to self-esteem, or guilt for the support receipt [45] which might negatively affect the quality of life. Other factors that might influence HRQoL among patients with visual impairment highlighted in the literature are age [12,35], gender [46], socioeconomic status [36], comorbidity [47], and use of eye care services [48].

The present study assessed visual outcomes and explored HRQoL and factors associated with it after cataract surgery among patients of Sevan Lions Regional Ophthalmic Unit (LROU) in Armenia. 

### Situation in Armenia

Armenia is a post-Soviet state with a population of 2.79 million, located in the South Caucasus [49]. After declaring independence in 1991, the economy has undergone intense transformation and the healthcare system in Armenia has been converted from a centrally run state system into a decentralized system with limited access to the population to specialized healthcare services and underutilization of primary care [50]. Public and private services are provided by independent, self- or mixed-financed healthcare facilities [50]. While the government is financing primary and emergency care, inpatient care, and most outpatient medicines are covered out-of-pocket by the population. In 2019, out-of-pocket expenditures constituted over 84% of the total health expenditure in Armenia [51]. Meanwhile, public expenditure on health as a percentage of GDP is below 1.5 [51]. The eye care system is characterized by excessive physical infrastructure and overcapacity in eye clinics located in the capital city of Yerevan and a lack of clinics and staff in rural areas [52]. The most recent available data show that the cataract surgical coverage rate is low (38.1% for people having visual acuity < 6/60) with financial and geographic barriers being the main reasons for underutilization [53]. 

Visual outcomes after cataract surgery in Armenia have been previously examined. An assessment of community needs for cataract intervention in the Gegharkunik region of Armenia using WHO developed Rapid Assessment of Cataract Surgical Services (RACSS) methodology [54] in 2003, identified poor visual outcomes following cataract surgery in 22.9% of patients [53], which exceeds WHO standards by almost four times. The latest study conducted in Armenia in 2009 assessed the outcomes of cataract surgery in LROU in the Gegharkunik region of Armenia in a small sample of patients aged 50 years and over and attempted to identify factors associated with borderline and poor outcomes following cataract surgery [55]. The proportion of good, borderline and poor outcomes in LROU in the study was 78.3%, 15.5% and 6.2%, respectively. Age and ophthalmic comorbidity were found to be associated with vision acuity after cataract surgery [55]. 

To date, no studies have explored HRQoL and associated factors after cataract surgery in Armenia. 

## 2. Materials and Methods

### 2.1. Recruitment/Data Collection

We conducted a cross-sectional interviewer-administered survey along with eye screenings among the patients who underwent cataract surgery at LROU. LROU is located in the Gegharkunik province of Armenia and mostly serves Gegharkunik and Tavush provinces, with few clients from Yerevan. The annual workload of the clinic reaches around 250 cataract surgeries, which are paid out of pocket or covered by the Government for socially vulnerable people. Patients having any type of cataract surgery in either one or both eyes in 2012–2013, living in Armenia, and having adequate cognitive ability were selected from the clinic’s rosters for participation in the study. All eligible patients from Gegharkunik and Tavush provinces and Yerevan were invited to their respective facilities for ophthalmic examination and interview. The data were collected between October 2014 and February 2015. Out of 460 eligible patients, 248 (54.0%) participated in the study. Out of the remaining 212 patients, 28 (6.1%) died, 31 (6.7%) refused to participate, 88 (19.1%) were not available (either were out of the country or did not answer the phone calls), and 65 (14.1%) did not attend the appointment.

For this study, two survey teams were employed, each composed of an ophthalmologist, an ophthalmic nurse, and an interviewer. Prior to conducting the survey, all teams underwent training where they were taught the necessary skills and techniques to carry out the survey. Periodical spot checks and questionnaire reviews were carried out to ensure the quality of the collected data.

### 2.2. Study Instrument and Eye Screening Procedures

Face-to-face interviews using a structured questionnaire lasted 15–20 min on average. Short-Form Health Survey (SF-36) measured health-related quality of life [56]. This instrument contains 36 items, measuring eight dimensions of health and wellbeing: “physical functioning,” “role limitation due to physical problems,” “bodily pain,” “general health perceptions,” “energy/fatigue,” “social functioning,” “role limitation due to emotional problems,” and “emotional wellbeing.” Each dimension was scored from 0 (worst possible health state) to 100 (best possible health state) by coding, summating, and transforming relevant item scores according to the scoring guideline [57]. The mean scores of SF-36 “physical functioning,” “role limitation due to physical problems,” “bodily pain” and “general health” scales were defined as physical health components, and the mean score of SF-36 “emotional wellbeing,” “role limitation due to emotional problems,” “social functioning,” and “energy/fatigue” scales were defined as mental health components [58]. 

To measure receiving social support, an eight-item modified Medical Outcomes Study Social Support Survey (mMOS-SS) questionnaire was used, which focused on tangible (instrumental) and emotional social support [59]. The following questions were included: If you needed it, how often is someone available, (1) to help you if you were confined to bed?, (2) to take you to the doctor if you need it?, (3) to prepare your meals if you are unable to do it yourself?, (4) to help with daily chores if you were sick?, (5) to have a good time with?, (6) turn to for suggestions about how to deal with a personal problem?, (7) who understands your problems?, (8) to love and make you feel wanted? The answer options of “never”, “seldom”, “sometimes”, “very often”, and “always” were used. 

Giving tangible and emotional social support was measured by the ten-item Two-Way Social Support Scale [60]. The following statements were included: (1) I am there to listen to other’s problems; (2) I look for ways to cheer people up when they are feeling down; (3) People close to me tell me their fears and worries; (4) I give others a sense of comfort in times of need; (5) People confide in me when they have problems; (6) I help others when they are too busy to get everything done; (7) I have helped someone with their responsibilities when they were unable to fulfill them; (8) When someone I lived with was sick I helped them; (9) I am a person others turn to for help with tasks; (10) I give financial assistance to people. The answer options were “never”, “seldom”, “sometimes”, “very often”, and “always”. 

The average score for each item was calculated and used to obtain a score for each subscale from zero to 100 in both instruments. The higher scores indicate a higher level of receiving and giving social support [60,61]. Other factors explored in the study included socio-demographic characteristics of the respondents, use of eye care services, smoking status and presence of comorbidities. 

All participants underwent detailed ophthalmologic screening examination, including measurements of visual acuity by the Golovin–Sivtsev chart [62], measurements of intraocular eye pressure (IOP) and dilated eye fundus examination. Eye screenings were carried out by two experienced ophthalmologists, a nurse and an interviewer. 

### 2.3. Analysis 

Simple linear regression explored bivariate associations between risk factors and physical and mental components of HRQoL. The variables that were found to be significantly associated with each component in simple regression analysis at the level of *p* ≤ 0.05 were included in the multivariable model [63]. Multicollinearity was checked between all independent risk factors included in the regression model. Statistical Package for the Social Sciences (SPSS) version 17.0 was used to analyze the data (SPSS Inc., SPSS Statistics for Windows, Version 17.0. Chicago, IL, USA). 

## 3. Results

The mean (± standard deviation) time after surgery in the sample was 1.64 ± 0.59 years, ranging from 0.9 to 3.2. The mean age of the participants was 72.0 ± 8.83, ranging from 47 to 90 years. Women constituted 52.0% of the sample. About 9.3% of the sample had university or higher education (>13 years), 14.9% had college (>10–≤13 years) education, and 75.8% had secondary (≤10 years) education. Almost 36% of the respondents reported having hypertension. Bone and joint diseases and heart disease were reported by 35.5% and 21.8% of respondents, respectively. Overall, 77.0% of the study participants had unilateral cataract surgery, while 23.0% had bilateral surgery. Visual acuity in the better-seeing eye was used for defining the outcome among those who had bilateral cataract surgery. About 77% of the study participants had good visual outcomes after cataract surgery, 14.9% had borderline visual outcomes, and 8.5% had poor visual outcomes. In total, 305 eyes were operated on, out of which 72.8% had good visual outcomes, while 17.7% had borderline outcomes. Poor visual outcomes were detected in 9.5% of the eyes. Eye diseases such as glaucoma, diabetic retinopathy, and age-related macular degeneration were present in 3.6%, 4.0%, and 12.5% of patients, respectively. 

### 3.1. HRQoL 

The mean (±standard deviation) composite SF-36 score for the study sample was 49.2 ± 23.5. The mean composite score for physical health was 50.8 ± 25.6, while the mean composite score for mental health was 49.9 ± 24.3. The mean composite scores among patients with good, borderline and poor visual outcomes were 51.7 ± 23.6, 38.4 ± 19.6 and 35.7 ± 19.2, respectively (Table 1). Pain, physical functioning, general health, role limitations due to emotional problems and role limitations due to physical health substantially differed across the visual outcome categories (Table 1). The highest mean scores were recorded for social functioning (78.1 ± 26.9), pain (66.0 ± 27.4) and physical functioning (58.6 ± 31.7). The lowest mean scores were obtained for role limitations due to physical health (33.9 ± 46.8) and role limitations due to emotional problems (34.0 ± 46.9). The scores for each item of the scale are presented in Supplementary Table S1.

### 3.2. Giving and Receiving Social Support 

The mean scores for receiving tangible and emotional social support were 76.9 ± 26.8 and 74.7 ± 29.6, respectively. The mean scores for giving tangible and emotional social support were 45.3 ± 33.8 and 68.6 ± 28.0, respectively (Supplementary Table S2). 

### 3.3. Factors Associated with HRQoL: Results of Linear Regression Analysis

In the simple linear regression analysis, age, gender, education, socioeconomic status, marital status, employment status, smoking status, visual outcome after cataract surgery, having non-communicable disease, and giving tangible and emotional support were significantly associated with SF-36 mental and physical component scores (Table 2). Receiving tangible support was associated with the physical component only in the simple regression model. 

Female gender, poorer socioeconomic status, having at least one non-communicable disease, and receiving tangible social support were significantly associated with a lower physical component score of HRQoL, while giving tangible support was associated with a higher physical component score in the adjusted model (Table 2). For the mental component, significant independent associations with socioeconomic status, presence of non-communicable diseases, and giving tangible support were observed.

## 4. Discussion

The present study explored HRQoL and factors associated with it about two years after cataract surgery in Armenian patients. The mean global score of SF-36 was 49.2 among the study participants, with the mean SF-36 scores of 49.9 for mental and 50.8 for physical health components. Our study findings are mostly consistent with the literature, yet we found a slightly higher SF-36 mean score for the physical health component in our sample. Hong et al. assessed patients’ short-term satisfaction with cataract surgery and HRQoL and reported that the mean SF-36 scores were about 50.0 for mental and 40.0 for physical health components among people aged over 65 with cataract surgery after two years of follow-up in Sydney, Australia [30], while Groessl et al. found that the mean mental and physical health component scores of SF-36 were 53.5 and 44.8, respectively, among people aged over 35 at six months after cataract surgery in the US [64].

We found that visual outcome after cataract surgery in the Armenian population was below the standards recommended by the WHO, with about 72.8% of eyes having good visual outcome after cataract surgery, 17.7% of eyes with borderline visual outcome, and 9.5% with poor visual outcome based on the WHO classification [7]. While the exploration of reasons for poor outcomes is beyond the scope of this study, developing an awareness of the magnitude of the issue is the first step in improving the results of the surgery [5]. Insight into the reasons can be gained if the individual surgeons and the hospitals incorporate performance monitoring and patient follow-up in their regular practice [5].

Our study found that patients with good visual outcomes had significantly higher mean physical and mental component scores than those with borderline and poor visual outcomes after cataract surgery in the unadjusted analysis; however, the variable lost significance in the adjusted analysis.

In our study, the mean SF-36 physical component score was significantly lower in women compared to men. Women might report lower levels of HRQoL than men regardless of visual acuity or health status. Studies conducted to assess gender differences in HRQoL in patients with different health problems found that women reported consistently worse HRQoL than men [46,65]. Possible reasons for reporting low levels of HRQoL by women could be restrictions in daily life, lower satisfaction with health outcomes, financial dependency, limited opportunities for leisure activities and limited social roles [66,67]. In the study by Hallert et al., which explored perceptions of HRQoL among men and women with celiac disease, women were seeking an emotionally oriented strategy to cope with the disease and expressed less satisfaction with health outcomes compared to men [66]. Other authors have noted that women have higher selective attention to their bodies and might be more conscious of their physical condition than men [68], which explains the observed link with the physical component in particular.

The presence of chronic non-communicable diseases was strongly associated with poorer physical and mental HRQoL in our study population. Several studies reported this association [47,69]. The study by van Nispen et al., conducted among visually impaired older patients from four Dutch hospitals, revealed that people who reported diabetes, chronic obstructive pulmonary disease or asthma, stroke, cancer, musculoskeletal and gastrointestinal conditions had lower quality of life scores compared to those who had not [47], while Sazlina et al. found that the presence of non-communicable diseases such as hypertension, type 2 diabetes, asthma, hyperlipidemia, coronary heart disease and osteoarthritis was significantly associated with a lower physical health score of SF-36 among people aged over 55 in Malaysia [69]. The presence of ocular comorbidities, such as glaucoma, age-related macular degeneration, and diabetic retinopathy, was not associated with HRQoL in our study, which might be explained by the small sample of such cases in our study.

Lower socioeconomic status had a significant association with poorer physical and mental HRQoL scores following cataract surgery in our sample. The link between different indicators of socioeconomic status and HRQoL has been established in the literature [70,71]. Interestingly, the association between socioeconomic status and HRQoL in our study was maintained after we controlled for the presence of chronic diseases. Several authors have reported that people with lower socioeconomic status tend to have lower levels of HRQoL than those with higher socioeconomic status regardless of their morbidity levels [70,72]. Lower socioeconomic status might result in poorer health by increasing the risk of disease onset and the risk of disability among those who have already developed the disease [70]. It has been also hypothesized that socioeconomic status might act through mechanisms independent of disease; for example, it might influence cardio-respiratory and locomotor fitness in physical functioning [70]. Other authors suggested that for the same level of morbidity, people with lower socioeconomic status might be more pessimistic in their self-assessment of health; possibly because they have fewer material and social resources to cope with their conditions [73].

The present study found that higher levels of received tangible social support were associated with lower physical component scores, while giving tangible support was associated with better physical and mental HRQoL in the adjusted analysis. While many authors explored the effect of receiving social support on health and well-being, the independent influence of giving social support has been less frequently examined. Providing support to others was shown to be beneficial to well-being, while receiving support was less important to well-being, except when received from a spouse or sibling in a study conducted among older adults in the US [74]. The author used identity theory to explain the findings, claiming that relying on support from others can negatively affect people’s sense of competence and lead to feelings of neediness and dependency. On the contrary, providing support might allow older adults to engage in socially productive behaviors, which might improve their sense of well-being [74]. The exploration of the neural basis of giving support showed that its health benefits might be explained by the caregiving-related inhibition of threat-related neural and physiological responding [75], which might explain the links we observed for both the physical and mental components of HRQoL. However, since our data are cross-sectional, the causal order of the association between support and HRQoL is unclear. It is possible that those with better physical and mental health are more likely to provide support to others, while those having poorer health status are more likely to seek and receive social support [76]. The detection of an inverse association between the physical component (and not the mental health component) and receiving social support in our study, seems to particularly support this hypothesis.

We would like to acknowledge several limitations of this study. First, we measured social support with scales which have not been validated for use in the Armenian population. Second, our sample was restricted to adults from one ophthalmic clinic in the Sevan region, which limited the generalizability of the study findings. In addition, those who did not attend the appointments or refused to participate in our study could have had poorer health status and visual outcomes and lower HRQoL scores than those who ended up in our sample. Finally, the cross-sectional design precluded exploring causal relationships between HRQoL and its possible predictors.

## 5. Conclusions

The visual outcomes after cataract surgery in Armenia remain lower than the WHO-recommended standards. Clinicians and administrators should consistently investigate and audit their performance to maximize the surgical visual outcome in Armenian patients. A focus on women, patients with poor socioeconomic status and those with non-communicable diseases is recommended to improve HRQoL outcomes. The use of longitudinal designs in future investigations may provide further insights into the links between social support and other predictors and HRQoL following cataract surgery.

## Figures and Tables

**Table 1:** Composite and subscale mean HRQoL scores in the study sample.

	All Patients, n = 248	Good Visual Outcome (VA ≥ 6/18), n = 190	Borderline ^a^ Visual Outcome (<6/18 ≥6/60), n = 30	Poor ^b^ Visual Outcome (<6/60), n = 14
Subscale	Mean ± SD ^c^			
Social functioning	78.1 ± 26.9	80.3 ± 25.3	70.0 ± 35.7	71.4 ± 25.2
Pain	66.0 ± 27.4	66.8 ± 26.9	64.7 ± 26.3	52.0 ± 30.2
Physical functioning	58.6 ± 31.7	61.8 ± 31.9	45.2 ± 26.5	40.7 ± 30.2
Emotional wellbeing	45.8 ± 26.7	46.9 ± 26.4	35.6 ± 25.1	43.7 ± 25.8
General health	44.7 ± 17.7	46.0 ± 18.0	38.0 ± 14.5	36.1 ± 10.2
Energy/fatigue	41.9 ± 22.1	43.4 ± 21.9	32.0 ± 18.6	37.1 ± 22.1
Role limitations due to emotional problems	34.0 ± 46.9	38.4 ± 48.2	21.1 ± 40.6	7.14 ± 26.7
Role limitations due to physical health	33.9 ± 46.8	38.8 ± 48.1	16.7 ± 37.9	7.14 ± 26.7
*Physical health component*	50.8 ± 25.6	53.4 ± 25.7	41.1 ± 21.5	34.0 ± 19.9
*Mental health component*	49.9 ± 24.3	52.3 ± 24.0	39.7 ± 23.4	39.8 ± 19.2
Total	49.2 ± 23.5	51.7 ± 23.6	38.4 ± 19.6	35.7 ± 19.2

^a^ Seven people who had borderline visual outcome after unilateral cataract surgery and have better vision in another eye were removed from the analysis; ^b^ Seven people who had poor visual outcome after unilateral cataract surgery and have better vision in another eye were removed from the analysis; ^c^ Standard deviation.

**Table 2:** The results of simple and multiple linear regression analysis of factors influencing physical and mental components of HRQoL.

	The Physical Component of HRQoL						The Mental Component of HRQoL					
	Unadjusted			Adjusted			Unadjusted			Adjusted		
	B ^a^	95% CI	*p*	B	95% CI	*p*	B	95% CI	*p*	B	95% CI	*p*
Age	−0.62	−0.97; −0.26	0.001	−0.14	−0.57; 0.29	0.525	−0.50	−0.84; −0.16	0.004	0.03	−0.41; 0.47	0.897
Gender												
*Male*	ref.			ref.			ref.			ref.		
*Female*	−14.7	−20.9; −8.58	<0.001	−11.2	−20.6; −1.88	0.019	−10.6	−16.6; −4.72	<0.001	−4.61	−14.2; 5.03	0.347
Education												
*Secondary (≤10 years)*	ref.			ref.			ref.			ref.		
*College/University/Higher (>10 years)* ^b^	9.97	2.59; 17.3	0.008	−0.70	−7.29; 5.88	0.833	11.9	5.00; 18.9	0.001	5.30	−1.45; 12.0	0.123
Socio-economic status												
*Lower than average*	ref.			ref.			ref.			ref.		
*Average and higher than average* ^c^	8.77	2.21; 15.3	0.009	10.4	4.63; 16.3	<0.001	11.9	5.82; 18.1	<0.001	11.3	5.39; 17.3	<0.001
Marital status												
*Married*	ref.			ref.			ref.			ref.		
*Divorced/Widowed*	−10.5	−17.2; −3.76	0.002	−1.44	−8.26; 5.37	0.676	−9.93	−16.3; −3.56	0.002	−2.20	−9.19; 4.80	0.536
*Single*	−6.78	−27.4; 13.8	0.518	−14.6	−33.9; 4.60	0.135	−10.8	−30.4; 8.71	0.275	−11.5	−31.2; 8.25	0.252
Employment status												
*Retired*	ref.			ref.			ref.			ref.		
*Employed*	17.5	7.97; 27.1	<0.001	−0.38	−10.5; 9.73	0.941	13.3	4.11; 22.5	0.005	−1.27	−11.7; 9.14	0.811
*Unemployed*	14.0	−1.21; 29.1	0.071	6.63	−8.34; 21.6	0.384	12.2	−2.31; 26.8	0.099	4.67	−10.7; 20.0	0.550
Smoking												
*Never smoker*	ref.			ref.			ref.			ref.		
*Ever smoker*	7.09	−1.11; 15.3	0.090	−8.87	−18.6; 0.88	0.074	6.70	−1.15; 14.5	0.094	−2.78	−12.8; 7.24	0.585
*Current smoker*	14.2	5.53; 22.8	0.001	−8.86	−19.3; 1.59	0.096	9.96	1.67; 18.2	0.019	−3.44	−14.2; 7.31	0.529
Current visual outcome after cataract surgery												
*Good (≥6/18)*	ref.			ref.			ref.			ref.		
*Borderline (<6/18–≥6/60)*	−12.2	−21.9; −2.59	0.013	−2.99	−11.5; 5.55	0.491	−12.6	−21.7; −3.39	0.007	−4.79	−13.5; 3.96	0.282
*Poor (<6/60)*	−19.4	−33.0; −5.81	0.005	−11.0	−22.5; 0.53	0.061	−12.4	−25.3; 0.54	0.060	−5.19	−17.0; 6.66	0.389
Glaucoma	−5.03	−22.1; 12.1	0.563	-	-	-	−5.26	−21.5; 11.0	0.524	-	-	-
Age-related macular degeneration	−1.49	−11.2; 8.20	0.762	-	-	-	−1.52	−10.7; 7.67	0.745	-	-	-
Diabetic retinopathy	−1.18	−17.5; 15.1	0.887	-	-	-	4.69	−10.8; 20.1	0.551	-	-	-
At least one chronic non-communicable disease ^d^												
	−20.8	−27.2; −14.5	<0.001	−15.9	−22.0; −9.88	<0.001	−12.6	−18.9; −6.36	<0.001	−9.77	−16.0; −3.56	0.002
Receiving social support												
*Receiving tangible support*	−0.16	−0.28; −0.04	0.009	−0.23	−0.34; −0.11	<0.001	−0.01	−0.12; 0.11	0.913	-	-	-
*Receiving emotional support*	−0.07	−0.18; 0.03	0.174	-	-	-	0.08	−0.02; 0.18	0.139	-	-	-
Giving social support												
*Giving tangible support*	0.32	0.23; 0.40	<0.001	0.25	0.14; 0.37	<0.001	0.31	0.23; 0.39	<0.001	0.23	0.11; 0.35	<0.001
*Giving emotional support*	0.17	0.06; 0.28	0.003	0.06	−0.08; 0.20	0.413	0.22	0.12; 0.33	<0.001	0.01	−0.12; 0.15	0.819

^a^ Unstandardized regression coefficient. ^b^ The study combined the “college” and “university and higher” responses on educational status in the analysis due to the limited number of cases in the “university and higher” group. **^c^** The study combined the “average” and “higher than average” responses on socioeconomic status in the analysis due to the limited number of cases in the “higher than average” group. ^d^ Hypertension, heart diseases, diabetes, lung diseases, gastrointestinal diseases, renal diseases, bone and joint diseases.

## Data Availability

The datasets used for the study are available from the corresponding author upon reasonable request.

## Supplementary Materials

The following supporting information can be downloaded at: https://www.mdpi.com/article/10.3390/healthcare11172429/s1, Table S1: SF-36 per-item scores. Table S2: Receiving and giving social support item scores for the study participants.
