# Supplementary material for: Health-Related Quality of Life after Cataract Surgery in Armenia: A Cross-Sectional Survey
Source: Healthcare (Basel). 2023 Aug 30;11(17):2429. doi: 10.3390/healthcare11172429 (PMC10487762; doi:10.3390/healthcare11172429)
Supplement: Supplementary file 1 [file healthcare-11-02429-s001.zip › Supplementary Table S1.pdf]

Supplementary Table S1. SF-36 per-item scores

SF-36	N	Mean ± SD
SF_36_item_1. General Health	248	26.9 ± 20.1
SF_36_item_2. General health compared to one year ago	245	38.8 ± 18.8
SF_36_item_3. Vigorous activities, such as running, lifting heavy objects, participating in strenuous sports	248	45.2 ± 37.6
SF_36_item_4. Moderate activities, such as moving a table, pushing a vacuum cleaner, bowling, or playing golf	248	55.4 ± 38.4
SF_36_item_5. Lifting or carrying groceries	248	59.5 ± 38.4
SF_36_item_6. Climbing several flights of stairs	248	53.2 ± 35.8
SF_36_item_7. Climbing one flight of stairs	248	54.8 ± 35.9
SF_36_item_8. Bending, kneeling, or stooping	248	53.8 ± 35.4
SF_36_item_9. Walking more than a mile	248	53.0 ± 37.1
SF_36_item_10. Walking several blocks	248	62.5 ± 36.9
SF_36_item_11_Walking one block	248	63.5 ± 36.7
SF_36_item_12_Bathing or dressing yourself	248	84.9 ± 29.5
SF_36_item_13. Cut down on the amount of time you spent on work or other activities	248	34.3 ± 47.5
SF_36_item_14. Accomplished less than you would like	248	32.7 ± 47.0
SF_36_item_15. Were limited in the kind of work or other activities	248	34.3 ± 47.5
SF_36_item_16. Had difficulty performing the work or other activities (for example, it took extra effort)	248	34.3 ± 47.5
SF_36_item_17. Cut down the amount of time you spent on work or other activities	248	34.3 ± 47.5
SF_36_item_18. Accomplished less than you would like	248	33.5 ± 47.3
SF_36_item_19. Didn't do work or other activities as carefully as usual	248	34.3 ± 47.5
SF_36_item_20. During the past 4 weeks, to what extent has your physical health or emotional problems interfered with your normal social activities with family, friends, neighbors, or groups?	248	77.6 ± 29.2
SF_36_item_21. How much bodily pain have you had during the past 4 weeks?	248	64.3 ± 28.7

SF_36_item_22. During the past 4 weeks, how much did pain interfere with your normal work (including both work outside the home and housework)?	248	67.8 ± 28.1
SF_36_item_23. Did you feel full of pep?	248	27.0 ± 29.8
SF_36_item_24. Have you been a very nervous person?	248	50.6 ± 36.6
SF_36_item_25. Have you felt so down in the dumps that nothing could cheer you up?	248	55.2 ± 36.6
SF_36_item_26. Have you felt calm and peaceful?	248	39.3 ± 28.0
SF_36_item_27. Did you have a lot of energy?	248	24.0 ± 30.4
SF_36_item_28. Have you felt downhearted and blue?	247	53.5 ± 29.7
SF_36_item_29. Did you feel worn out?	247	60.7 ± 31.2
SF_36_item_30. Have you been a happy person?	248	30.3 ± 30.8
SF_36_item_31. Did you feel tired?	248	56.2 ± 25.1
SF_36_item_32. During the past 4 weeks, how much of the time has your physical health or emotional problems interfered with your social activities (like visiting with friends, relatives, etc.)?	248	78.5 ± 29.1
SF_36_item_33. I seem to get sick a little easier than other people	248	61.6 ± 30.2
SF_36_item_34. I am as healthy as anybody I know	248	53.1 ± 17.5
SF_36_item_35. I expect my health to get worse	248	54.7 ± 25.3
SF_36_item_36. My health is excellent	248	27.4 ± 32.3
