# Supplementary material for: Health-Related Quality of Life after Cataract Surgery in Armenia: A Cross-Sectional Survey
Source: Healthcare (Basel). 2023 Aug 30;11(17):2429. doi: 10.3390/healthcare11172429 (PMC10487762; doi:10.3390/healthcare11172429)
Supplement: Supplementary file 1 [file healthcare-11-02429-s001.zip › Supplementary Table S2.pdf]

Supplementary Table S2. Receiving and giving social support item scores for the study participants

	N	Mean ± SD
**Receiving Social Support**		
If you need it, how often is someone available…		
Item 1. to help you if you were confined to bed?	248	77.6 ± 27.4
Item 2. to take you to the doctor if you need it?	248	71.9 ± 32.7
Item 3. to prepare your meals if you are unable to do it yourself?	248	81.6 ± 26.3
Item 4. to help with daily chores if you were sick?	248	76.5 ± 29.2
**Receiving tangible support (Item 1-4) – total score**	**248**	**76.9 ± 26.8**
Item 5. to have a good time with?	248	75.5 ± 29.7
Item 6. turn to for suggestions about how to deal with a personal problem?	248	74.1 ± 30.5
Item 7. who understands your problems?	248	74.4 ± 30.7
Item 8. to love and make you feel wanted?	248	74.7 ± 31.4
**Receiving emotional support (Item 5-8)– total score**	**248**	**74.7 ± 29.6**
**Giving Social Support**		
Item 1. I am there to listen to other's problems.	248	69.1 ± 29.3
Item 2. I look for ways to cheer people up when they are feeling down	248	68.2 ± 29.5
Item 3. People close to me tell me their fears and worries.	248	67.9 ± 29.2
Item 4. I give others a sense of comfort in times of need.	247	68.2 ± 28.9
Item 5. People confide in me when they have problems.	248	68.2 ± 29.5
**Giving emotional support (Item 1-5) – total score**	**247**	**68.6 ± 28.0**
Item 6. Instrumental support: I help others when they are too busy to get everything done.	248	47.4 ± 35.7
Item 7. I have helped someone with their responsibilities when they were unable to fulfill them.	248	45.9 ± 36.4
Item 8. When someone I lived with was sick I helped them.	247	55.5 ± 36.1
Item 9. I am a person others turn to for help with tasks.	248	47.3 ± 35.3
Item 10. I give financial assistance to people in my life.	248	31.0 ± 38.5
**Giving tangible support (Item 6-10)– total score**	**247**	**45.3 ± 33.8**
